# Torin2 inhibits the EGFR-TKI resistant Non-Small Lung Cancer cell proliferation through negative feedback regulation of Akt/mTOR signaling

**DOI:** 10.7150/jca.37417

**Published:** 2020-07-29

**Authors:** Yi Hu, Ji Zhang, Qun Liu, Mingyao Ke, Jiurong Li, Wenhao Suo, Weixi Guo, Aiping Ma

**Affiliations:** 1 Department of Clinical Laboratory, The first affiliated hospital, School of Medicine, Xiamen University, Xiamen, China.; 2 Department of Respiratory and Critical Medicine, The first affiliated hospital, School of Medicine, Xiamen University, Xiamen, China.; 3 Wuxi Lung Transplant Center, Wuxi People's Hospital affiliated to Nanjing Medical University, Wuxi, China.; 4 Department of Pathology, The first affiliated hospital, School of Medicine, Xiamen University, Xiamen, China.; 5 Department of Thoracic Surgery, The first affiliated hospital, School of Medicine, Xiamen University, Xiamen, China.; 6 Department of Respiratory and Critical Medicine, The secondary hospital of Xiamen Medicine school, Xiamen, China.

**Keywords:** mTOR, Torin2, EGFR-TKI, Autophagy, Erlotinib

## Abstract

It is known that mammalian target of rapamycin (mTOR) signaling plays an important role in NSCLC cells proliferation. Torin2 is a second-generation ATP-competitive inhibitor which is selective for mTOR activity. In this study, we investigated whether torin2 was effective against lung cancer cells, especially EGFR-TKIs resistant NSCLC cells.

We found that torin2 dramatically inhibited EGFR-TKI resistant cells viability *in vitro*. In xenograft model, torin2 treatment significantly reduced the volume and weight of xenograft tumor in the erlotinib resistant PC9/E cells. Additionally, autophagy protein of phosphatidylethanolamine-modified microtubule-associated protein light-chain 3II/I (LC3II/I) increased in PC9/E after torin2 treatment. Torin2 blocked the level of phosphorylated S6 and the phosphorylation of Akt at both T308 and S473 sites compared with erlotinib treatment. Furthermore, TUNEL assay showed that apoptosis of tumor tissue increased significantly in the torin2 treatment group. Immunohistochemical analysis demonstrated that tumor angiogenesis was obviously inhibited by torin2 treatment in EGFR-TKI resistant group. Collectively, our results suggested that torin2 could inhibit the NSCLC cells proliferation by negative feedback regulation of Akt/mTOR signaling and inducing autophagy. This suggests that torin2 could be a novel therapeutic approach for EGFR-TKI resistant NSCLC.

## Introduction

Epidermal growth factor receptor (EGFR) is the main oncogenic driver in non-small cell lung cancer (NSCLC) and is overexpressed in 62% of NSCLC cases 1. The epidermal growth factor receptor tyrosine kinase inhibitors (EGFR-TKIs) are effective targeted therapies for advanced NSCLC patients with EGFR exon 19 deletions or an exon 21 L858R mutation 2, 3. Despite the initial success of different generation EGFR inhibitors, secondary resistance inevitably emerges in majority of patients over time 4. Several mechanisms of EGFR-TKIs acquired resistance are involved in progression of NSCLC, including T790M mutation, upregulation of MET/HGF, HER2 mutations, overexpression of HER3, persistent activation of IGF-1R, mutations of PIK3CA/AKT, and abnormal dimerization of STAT3 5, 6. However, since nearly 30% of the acquired resistance cannot be explained with the recognized mechanisms, some unknown mechanisms remain needed to be investigated 7. It has been reported that the mammalian target of rapamycin (mTOR) pathway was abnormally activated in NSCLC as evidenced by phosphorylation of mTOR (69%), p70 S6K (81%), and 4EBP-1 (79%) in tumor tissue 8, 9. mTOR is a serine-threonine kinase belonging to the phosphatidylinositol 3-kinase (PI3K) family of proteins that controls cell survival and growth by regulating various cellular process including protein synthesis and autophagy 10, 11. mTOR protein complexes have two important components of mTOR complex 1 (mTORC1) and mTORC2, both of which are involved in negative feedback loops which modulate signaling. mTORC1 is responsive to rapalogues and directly activates eukaryotic translation initiation factor 4EBP1 and p70 S6K to facilitate translation, whereas mTORC2 is relatively insensitive to rapalogues and induces the phosphorylation of Akt at serine 473 12-15.

Activation of the PI3K/Akt/mTOR is closely associated with EGFR-TKIs resistance 16. Cheng et al. indicated that long non-coding RNA UCA1 induced non-T790M acquired resistant lung cancer cells, in which the mechanism was associated with activation of Akt/mTOR pathway 17. Pirazzoli V and colleagues demonstrated that mTORC1 was activated in EGFR-mutant lung adenocarcinomas 18. Bianco et al. showed that combination of mTOR inhibitor everolimus and gefitinib inhibited growth of gefitinib resistant colon cancer xenografts 19. However, many recent studies have also highlighted that single-agent PI3K pathway inhibitors may not be effective for EGFR-driven lung cancers 20, 21. Previous data showed that the combination of PI3K-mTOR and MEK inhibitors effectively shrunk tumors in a transgenic and xenograft model of EGFR T790M-L858R cancers 22, 23. Although inhibiting PI3K/Akt/mTOR pathway has emerged as a possible solution to EGFR inhibitor resistance in many preclinical studies, results have demonstrated that mTORC1 inhibitor, rapamycin, causes phosphorylation of Akt through inhibition of the p70 S6K feedback loop to limit the availability 24, 25. Therefore, it is necessary to screen more efficient methods that can improve the clinical outcome of patients with aberrant activation of mTOR.

Torin2 is a second-generation ATP-competitive mTOR inhibitor and also a potent inhibitor of serine/threonine kinases ATR, ATM and DNA-PK. It is reported that torin2 can inhibit proliferation, and induce apoptosis and autophagy of cancer cells by reducing mTORC1 and mTORC2 activity 26, 27. Several preclinical studies have reported that torin2 could inhibit proliferation and migration in hepatocarcinoma, thyroid cancer and epithelial ovarian cancer 28-30. However, it is not known whether torin2 could suppress EGFR-TKIs resistant lung cancer.

In the study, we found that torin2 significantly inhibited the proliferation of EGFR-TKIs resistant NSCLC* in vitro* and *in vivo*. Torin2 reduced the activity of Akt/mTOR signaling and induced the autophagy compared with erlotinib treatment. It was suggested that torin2 might be a clinically useful therapeutics for EGFR resistant lung cancer.

## Materials and Methods

### Cell lines and cell culture

Human lung cancer cell lines A549, H1650, H1975 and HBE were purchased from the American Type Culture Collection (ATCC, Manassas, VA). The cells were cultured in RPMI 1640 (Gibco, Carlsbad, CA, USA) medium and supplemented with 10% fetal bovine serum (FBS) (Invitrogen, Inc., Carlsbad, CA, USA), 50 U/mL penicillin, and 50 mg/mL streptomycin (Sigma-Aldrich, Inc., Poole, Dorset, UK), at 37°C in a humidified incubator with 95% air and 5% CO_2_ by volume. Cells were sub-cultured or plated for subsequent experiments until they approached approximately 80% confluence. HBE was derived from normal human bronchial epithelial cell. A549 cells harbor KRAS G12S mutation and EGFR wild type. PC9 cells harboring an EGFR exon 19-frame deletion were sensitive to erlotinib. H1650 cells harboring an EGFR exon 19-frame deletion and PTEN loss were resistant to erlotinib. H1975 cells carrying the EGFR L858R-T790M mutation were resistant to erlotinib. PC9/E acquired erlotinib resistance following chronic exposure of PC9 cells to medium with increasing erlotinib concentrations. PC9 cells were exposed to 2 μM erlotinib in medium containing 10% FBS, and the concentration was increased in a stepwise manner. Cells that were able to grow in 50 μM erlotinib were obtained 4 months after the initial exposure 31, 32.

### Cell viability assay

The Cell Counting Kit-8 reagent (CCK-8) (Dojindo Molecular Technologies, Inc., Kumamoto, Japan) was used to assess cell proliferation. Cells were seeded in 96 well plates at approximately 4000 cells per well with 100 μl of culture medium. After 24 hours, 10 μl of CCK-8 solution was applied to each well, and the plates were incubated for 1 hour at 37°C. Finally, the light absorbance values at 450 nm were determined using a microplate reader (Multiskan, Thermo Fisher Scientific, Inc., Waltham, MA, USA). Data were calculated as the percentage of the total absorbance of treated cells/absorbance of non‐treated cells and were expressed as the mean ± standard error of the mean.

### Western blot

Cells were washed with PBS and harvested after 3 days of culture. Cells were lysated for 30 min on ice in RIPA buffer (Sigma-Aldrich, St. Louis, MO, USA) in the presence of a cocktail proteinase inhibitor (Sigma-Sigma-Aldrich, St. Louis, MO, USA). Cell lysates were centrifuged at 2000 rpm, 4°C. The protein lysates were separated using SDS-PAGE and transferred to a PVDF (Millipore, Billerica, MA, USA) membrane. The membrane was blotted with specific primary and horseradish peroxidase (HRP) secondary antibodies. Antibody binding was detected by enhanced chemiluminescence detection kit (ECL) (Amersham International Plc., Buckinghamshire, UK). The following antibodies were used: BAX (#5023), BCL-2, (#4223), cleaved PARP (#5625), anti-Akt (#2920) and phospho-Akt at Ser473 (#9271), 4EBP-1 (#9459) and phospho-4EBP-1 (#2855), β-actin (#3700), anti-S6 (#2317) and phospho-S6 at Ser240/244 (#5364) (diluted with 5% BSA to 1: 1000) were obtained from Cell Signaling Technology, Beverly, MA, USA). p62/SQSTM1 (#H00008878-M01) from Abnova (Taipei City, Taiwan), and LC3 (L8918) and β-actin (#A5316) from Sigma-Aldrich (St. Louis, MO, USA).

### Patient samples and immunohistochemical staining

Human lung cancer tissues (including 5 NSCLC samples) and adjacent normal tissues were fixed with 4% paraformaldehyde and embedded in paraffin for sectioning. Patients underwent surgery at The First Affiliated Hospital of Xiamen University during the period from 2016 through 2017. Antibody for Phosphor-S6 (#4858) (Cell Signaling Technology, Beverly, MA, USA) was used in immunohistochemical staining (IHC) analysis. The Ethical Committee of The First Affiliated Hospital of Xiamen University approved the tumor tissues. The degree of immunostaining of paraffin-embedded sections was scored independently by two observers, based on the intensity index of staining. The proportion of tumor cells was scored as follows: 1 (< 10% positive tumor cells), 2 (10%-50% positive tumor cells), and 3 (> 50% positive tumor cells). The intensity of staining was graded according to the following criteria: - (no staining); + (weak staining = light yellow), ++ (moderate staining = yellow brown), and +++ (strong staining = brown).

### Apoptosis assay

Apoptosis was measured with TUNEL staining. Apoptotic cells in the PC9/E xenograft tumors were identified by terminal deoxynucleotidyl transferase-mediated dUTP nick end-labeling (TUNEL) staining using the *In Situ* Cell Death Detection Kit (Roche Applied Science, Mannheim, Germany) according to the manufacturer's instructions. The tissue sections were deparaffinized before rehydration with decreasing concentrations of ethanol. After washing with 0.85% NaCl and PBS, the tissue sections were fixed with 4% formaldehyde for 15 min. Following washing with PBS, the tissue sections were covered with proteinase K solution for 15 to 20 min. After another PBS wash, the tissue sections were covered with the TUNEL reaction mixture and incubated for 1 h in the dark. DAPI counterstaining was followed by a final PBS wash, and tissue sections were then examined and photographed using Olympus microscopy.

### Reagents

Rapamycin and erlotinib were purchased from Selleck Chemicals (Houston, TX, USA). Torin2 was also purchased from Selleck Chemicals (Houston, TX, USA) for *in vitro* and *in vivo* experiments. Torin2 was diluted in dimethyl sulfoxide (DMSO) for *in vitro* studies.

### Xenograft studies

Female athymic BALB/c nude and Nodscid mice (Beijing Institute of Material Medicine, Chinese Academy of Science, China) were maintained in a specific pathogen-free facility. Animal handling and procedures were approved by the Xiamen University Institutional Animal Care and Use Committee. PC9/E (2.5×10^6^ cells) and H1975 (5.0×10^6^ cells) cells were subcutaneously inoculated into the flank region of 6-week-old female mice. PC9/E cells were transplanted into BALB/c nude mice (n=5 per group). H1975 cells were transplanted into Nodscid mice (n=7 per group). Eighteen days later, Torin2 (10 mg/kg) was intraperitoneally injected into the nude mice every three days. Tumor sizes were calculated with the formula: (mm^3^) = 0.5 × *a* × *b*^2^, in which *a* is the largest diameter and *b* is the perpendicular diameter. The tumor volumes were measured every other day. After 3 weeks, all mice were sacrificed and tumors were dissected out. Survival was recorded daily.

### Immunohistochemical staining of xenograft tissue

Fresh tumors were removed from each group mice and fixed for 24h in 4% paraformaldehyde, embedded in paraffin, sliced into 5μm thick sections. Immunohistochemical (IHC) staining was performed using standard procedures. The slides were deparaffinized through a series of graded alcohol. High-temperature antigen retrieval was performed in a citrate salt antigen repair solution for 10 min. Then the slides were incubated in blocking serum for 30 min. Primary anti-CD31 antibody (1:100, rat monoclonal, #77699, Cell Signaling Technology, Danvers, MA, USA) was applied. The slides were incubated overnight at 4 °C. After repeated washing, the slides were incubated with biotinylated goat-anti-rat secondary antibody (1:200, goat polyclonal, #2091, Santa Cruz Biotechnology, Dallas, Texas, USA) and further incubated with streptavidin-horseradish peroxidase complex for 20 min. Sections were finally stained with diaminobenzidine and counterstained with hematoxylin and eosin. The area of positive staining was measured in five different images taken at 200× magnification on each slide and quantified using Image Pro Plus 6.0 software.

### Statistical analysis

All data were expressed as mean ± SEM. Statistical analysis was performed by using SPSS 17.0 software. Student's t test was used for the two-group comparisons, while one-way ANOVA was used for comparisons among multiple groups. A *P*-value less than 0.05 was considered statistically significant.

## Results

### The mTORC1 pathway abnormally activated in non-small cell lung cancer

**Figure 1:**
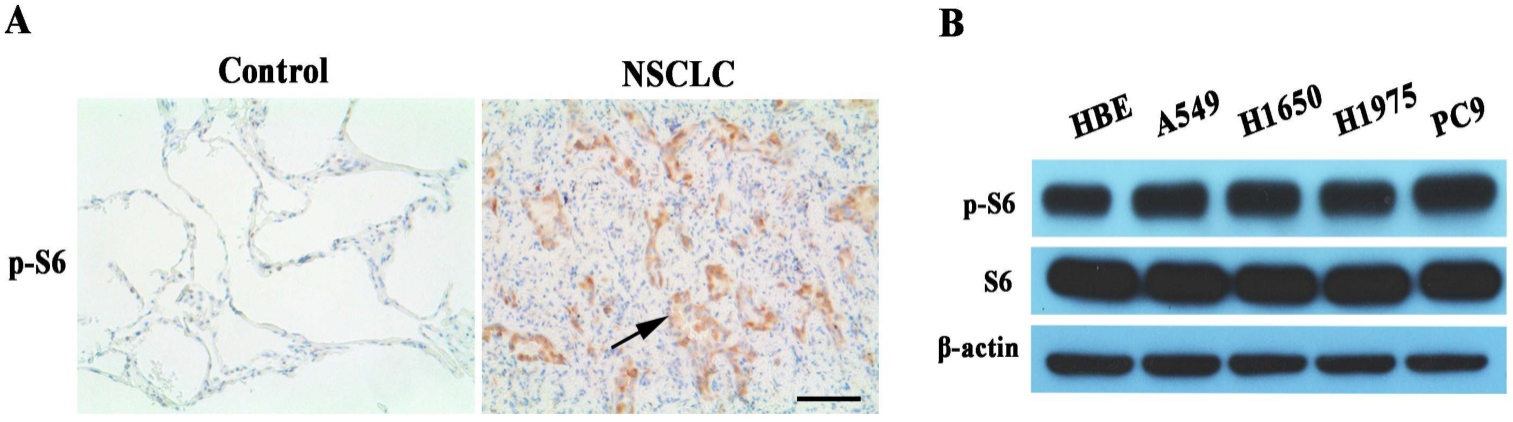
** Assessment of mTORC1 activity in NSCLC. (A)** Representative microphotographs of Phosphor-S6 expression in EGFR-TKI resistant NSCLC and para-cancerous tissues (Control). High expression levels of Phosphor-S6 was found in lung cancers (NSCLC), whereas the expression was low in lung cancer para-cancerous tissues (Control). 200x magnification, Scale bar:100μm. The arrow is presented as lung cancer cells. **(B)** Relative expression levels of phosphor-S6 protein were analyzed in HBE and 4 different lung cancer cell lines by Western blot. High expression levels of Phosphor-S6 was found in lung cancer cell lines.

We examined phosphor-S6 protein expression of mTORC1 signaling pathway in human lung cancer tissues. Phosphor-S6 protein overexpression was observed in EGFR-TKI sensitive or resistant lung cancer tissues with IHC analysis (Figure 1A). Also, the phosphor-S6 expression level of mTORC1 was analyzed by Western blot in a panel of five kinds of cell lines, among which A549, H1650 and H1975 are EGFR-TKI resistant. HBE is normal human bronchial epithelial cell line, and PC9 is EGFR-TKI sensitive. The result indicated that phosphor-S6 protein was up-regulated in lung cancer cell lines compared with normal lung epithelial cells, as shown in Figure 1B.

**Figure 2:**
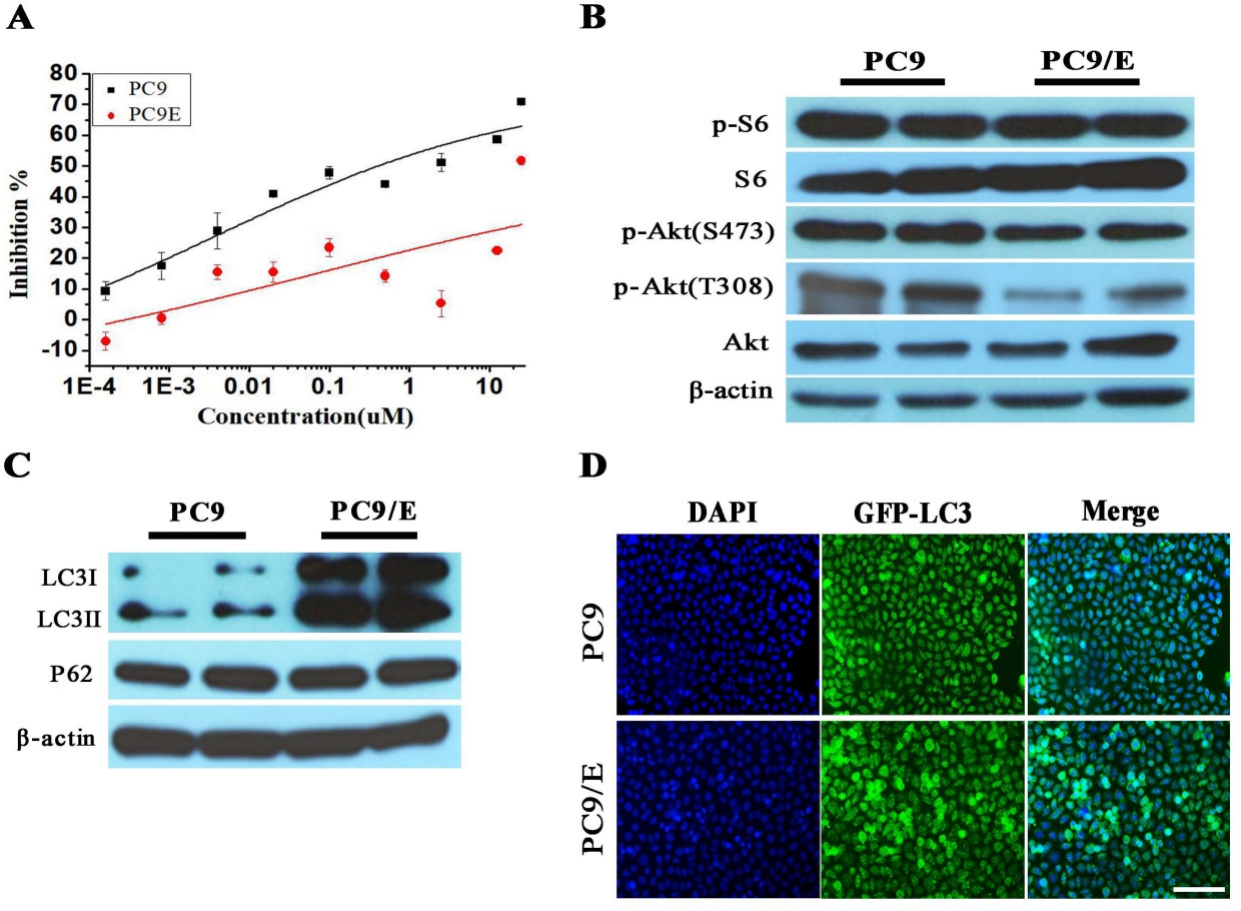
** Characteristics in Erlotinib-resistant NSCLC cell line. (A)** Erlotinib-resistant NSCLC cell line PC9/E was established. **(B)** The high level of phosphorylated S6 was observed in PC9 and PC9/E cells, but phosphorylated Akt (S473) and Akt (T308) were decreased in PC9/E compared to PC9. **(C)** The expression of LC3II/I protein was induced in PC9/E cell compared with PC9. There was no significant change in P62 protein expression between PC9 and PC9/E cell. **(D)** Under the fluorescence microscope, DAPI of blue fluorescence represents cell nucleus, GFP-LC3 dot formation of green fluorescence represents autophagic vacuoles. Induced autophagy was observed in PC9/E cell compared with PC9 cell. Scale bar: 150μm.

To further confirm the abnormal expression of phosphor-S6, we established the erlotinib-resistant NSCLC cell line PC9/E from the erlotinib-sensitive PC9 cell line. Erlotinib-resistant PC9/E cell was developed by continuous exposure of erlotinib-sensitive PC9 cells to sequentially increasing concentrations of erlotinib. It was found that the cell viability of the PC9 cells was dramatically reduced when the cells were incubated with 0.1 μM erlotinib for 72 hours, whereas the viability of the PC9/E cells was not significantly decreased by up to 10 μM erlotinib treatment (Figure 2A). Furthermore, the level of phosphorylated S6 was obviously enhanced in PC9 and PC9/E cell, but the expression of phosphorylated Akt at S473 and T308 sites was decreased in PC9/E compared with PC9 cell (Figure 2B). It was also found that the expression of autophagy protein LC3II/I was dramatically increased in PC9/E cell (Figure 2C). With immunofluorescent staining, the accumulation of green fluorescent protein-LC3 punta was observed in PC9/E cell compared with PC9 cell, as shown in Figure 2D. Collectively, the results showed that mTORC1 was activated in the in EGFR TKI-sensitive and resistant NSCLC Cell lines and autophagy was induced in EGFR-TKI resistance PC9/E.

### Torin2 reduce EGFR-TKI resistant cells viability

**Figure 3:**
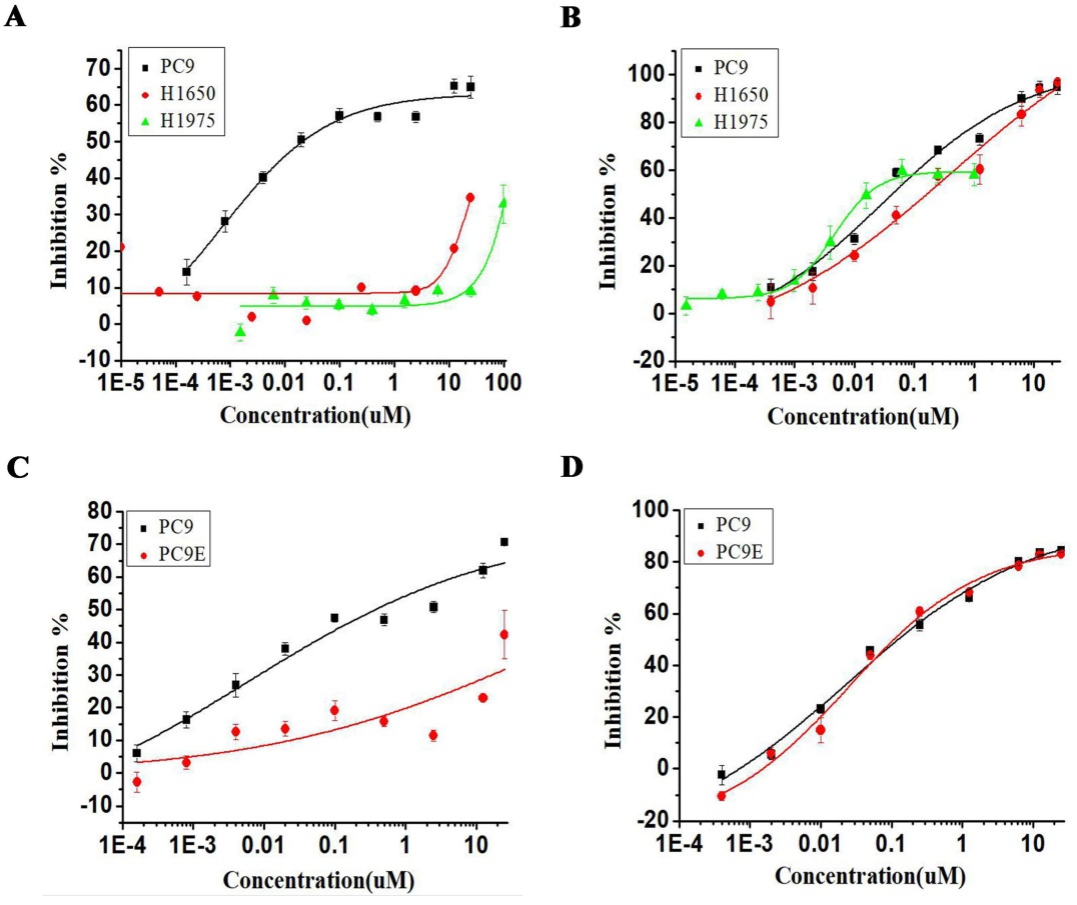
** Inhibitory effect of Torin2 on human NSCLC PC9/E cell line.** Cell viability was measured using the CCK8 assay. **(A)** Erlotinib dramatically suppressed the PC9 cell viability but not H1975 and H1650 cell lines. **(B)** Torin2 obviously inhibited cell viability of H1975, PC9, H1650 cell line. **(C)** PC9/E cells were treated with Erlotinib for 72h, respectively. Erlotinib obviously decreased the PC9 cell viability compared to PC9/E cell. **(D)** The cell viability of PC9/E and PC9 cell were apparently repressed by Torin2 treatment.

We next investigated the effect of torin2 treatment on EGFR-TKI resistant cell lines. As shown in Figure 3A, erlotinib treatment decreased the PC9 cell viability but showed no effect on the growth of H1975 and H1650 cell lines. In contrast, torin2 treatment markedly inhibited the cell growth of EGFR-TKI resistant cell lines, H1975, H1650 and sensitive PC9 cell line, as measured with CCK-8 assay (Figure 3B). Also, erlotinib failed to reduce the growth of PC9/E compared with PC9 (Figure 3C), while there was a dramatic inhibition in PC9/E and PC9 cells viability by torin2 treatment (Figure 3D). The results showed that torin2 had a marked inhibition effect on EGFR-TKI resistant cells viability.

### The inhibitory effect of torin2 on the EGFR-TKI resistance cells *in vivo*

**Figure 4:**
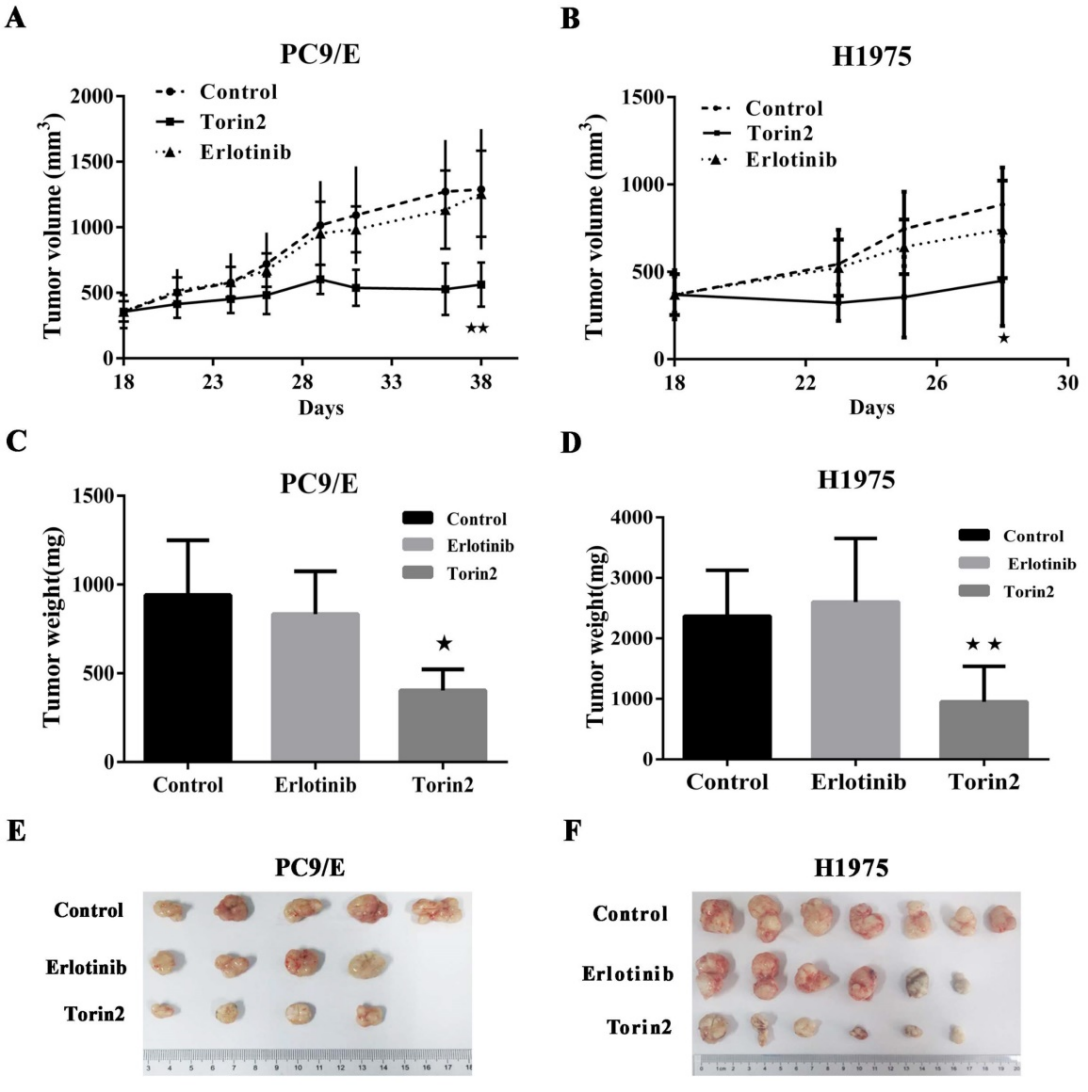
** Effects of Torin2 on the Erlotinib-resistant NSCLC cell lines growth *in vivo*. (A)** Tumor size was measured in each group (n=5) injected by PC9/E every 2 days for indicated period. Data are expressed as the mean ± SD. **(B)** H1975 cells were transplanted into Nodscid mice (n=7 per group). The changes of tumor volume were observed, ★p<0.05. **(C)** Tumor weight was measured from nude mice transplanted with PC9/E in each treatment group cell. There was a significant decrease in tumor weight from Torin2 treated group. **(D)** Tumor weight was recorded in each group from Nodscid mice injected with H1975. The tumor weight of Torin2 treated group significantly decreased compared with other treatments, ★★p<0.01. **(E)** The representative tumors were shown from nude mice in each treatment group. **(F)** Tumors were from Nodscid mice in each treatment group.

The promising *in vitro* findings encouraged us to further investigate whether torin2 treatment could suppress EGFR-TKI resistance cells *in vivo*. To do this, we generated xenograft tumors by subcutaneous injection of PC9/E cells into BALB/c nude mice and then treated those mice with torin2 or erlotinib. Eighteen weeks after the transfer of PC9/E line, the volume of tumors from the control mice was 853.2 ± 127.7 mm^3^. The volume of tumor in erlotinib treated mice had no significance difference compared the control group (807.3 ± 112.9 mm^3^ vs 853.2 ± 127.7 mm^3^, P=0.79). The recipient mice treated with torin2 displayed a significantly smaller xenograft tumor (491.9 ± 28.9 mm^3^), than mice treated with erlotinib (491.9 ± 28.9 mm^3^ vs 807.3 ± 112.9 mm^3^, P=0.017, Figure 4A). Moreover, the inhibition effect of torin2 was confirmed by in another xenografted tumor model in Nodscid mice induced by the transfer of H1975 cells. There was no difference between the erlotinib group and control group in volume tumor (763.8 ±155.8 mm^3^ vs 919.2 ± 221.8 mm^3^, P=0.58). The tumor size of mice treated with torin2 was markedly decreased than erlotinib group (374.3 ± 26.95 mm^3^ vs 763.8 ± 155.8 mm^3^, P=0.042, Figure 4B).

In addition to the volume of tumor, the weight of tumor in mice treated with torin2 was also significantly less than that of mice treated with erlotinib after injection of PC9/E line (403 ± 59.35 vs 832.5 ± 120.3 mg, P=0.0185). No difference was found in the weight of tumor between erlotinib group and control group (832.5 ± 120.3 vs 940 ± 138.2 mg, P=0.587, Figure 4C). Thus, these results suggested an inhibition effect of torin2 on EGFR-TKI resistance cells *in vivo* tumor models. In H1975 line, there was no significance difference between the erlotinib group and control group with regard to the weight of tumor (2370 ± 465.8 vs 2766 ± 353.2 mg, P=0.51, Figure 4D). But the weight of tumor in torin2 treated group was dramatically reduced compared with erlotinib group (770 ± 301 vs 2370 ±465.8mg, P=0.01, Figure 4D). PC9/E was subcutaneously injected in to the BALB/c nude mice. Tumors from sacrificed mice were dissected 21 d after subcutaneous injection and are shown in the indicated row representatively. As shown in Figure 4E, xenograft tumor growth was reduced in the Torin2 group as compared with the control group and erlotinib group. Also, H1975 cells were xenografted into Nodscid mice. Torin2 was found to drastically repress tumor growth when compared with the control group and erlotinib group (Figure 4F).

### Torin2 induced autophagy in NSCLC cell lines

**Figure 5:**
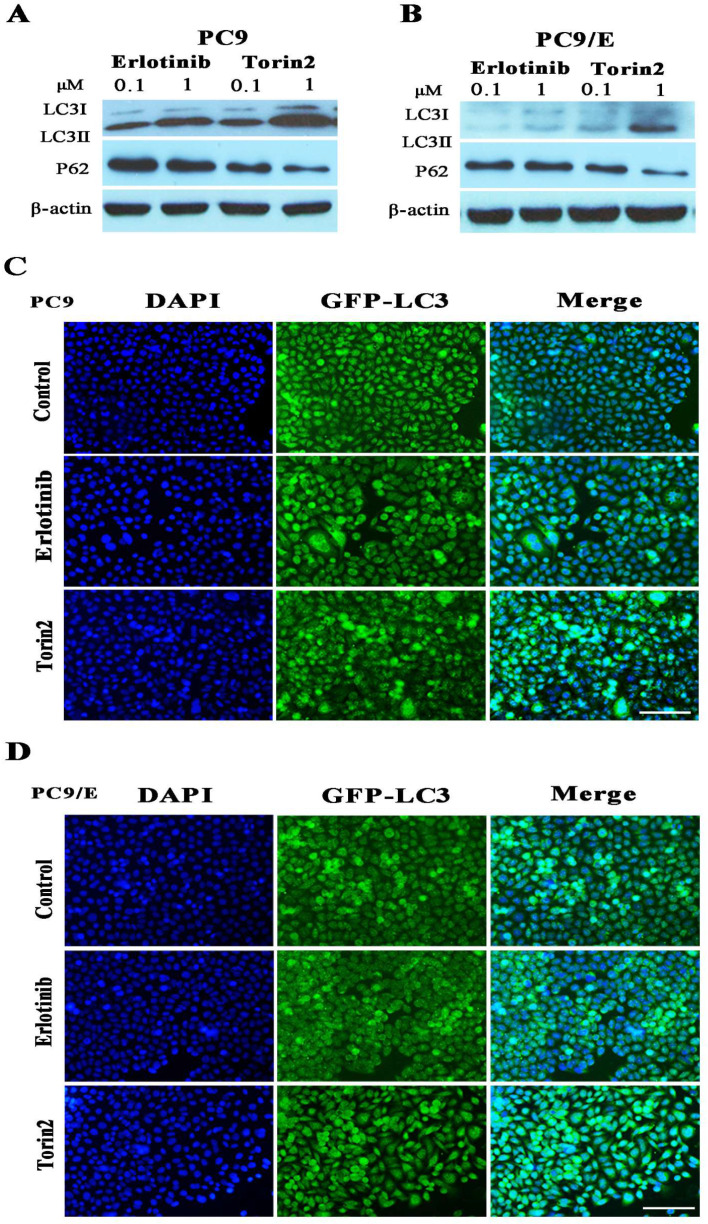
** Torin2 induced autophagy in NSCLC cells. (A)** Cells were incubated with the indicated concentration of erlotinib, torin2 for 24 hours. The level of LC3II/I was significantly increased in a dose-dependent manner after 24 hours of exposure to erlotinib or torin2 in PC9 cell line. **(B)** The expression of LC3-II was much higher in the PC9/E cell line treated by torin2. Also, the levels of P62 protein decreased respectively in PC9 and PC9/E cell lines treated with torin2 compared with erlotinib. **(C)** Induced-autophagy was analyzed by immunofluorescent microscope, autophagy was enhanced by erlotinib or torin2 treatment in PC9, Scale bar: 100 µm. **(D)** Autophagy was increased by erlotinib or torin2 treatment in PC9/E. Scale bar: 100 µm.

To further explore the mechanism behind the inhibition effect of torin2 on EGFR-TKI resistance cells, we determined cell autophagy using Western blot. It revealed that autophagy protein LC3II/I was induced in PC9 cell treated by torin2 or erlotinib in a dose dependent manner (Figure 5A). However, torin2 induced significantly higher expression of LC3II/I protein in PC9/E cell line than erlotinib (Figure 5B). The levels of P62 protein decreased respectively in PC9 and PC9/E cell line treated with torin2 compared with erlotinib. Furthermore, as shown in Figure 5C and 5D, torin2 induced GFP-LC3 autophagy expression in both PC9 and PC9/E cells. Taken together, the results demonstrated that torin2 induced autophagy in both PC9 and PC9/E cell.

### Torin2 inhibited cell proliferation through down-regulated negative feedback of mTOR signaling

**Figure 6:**
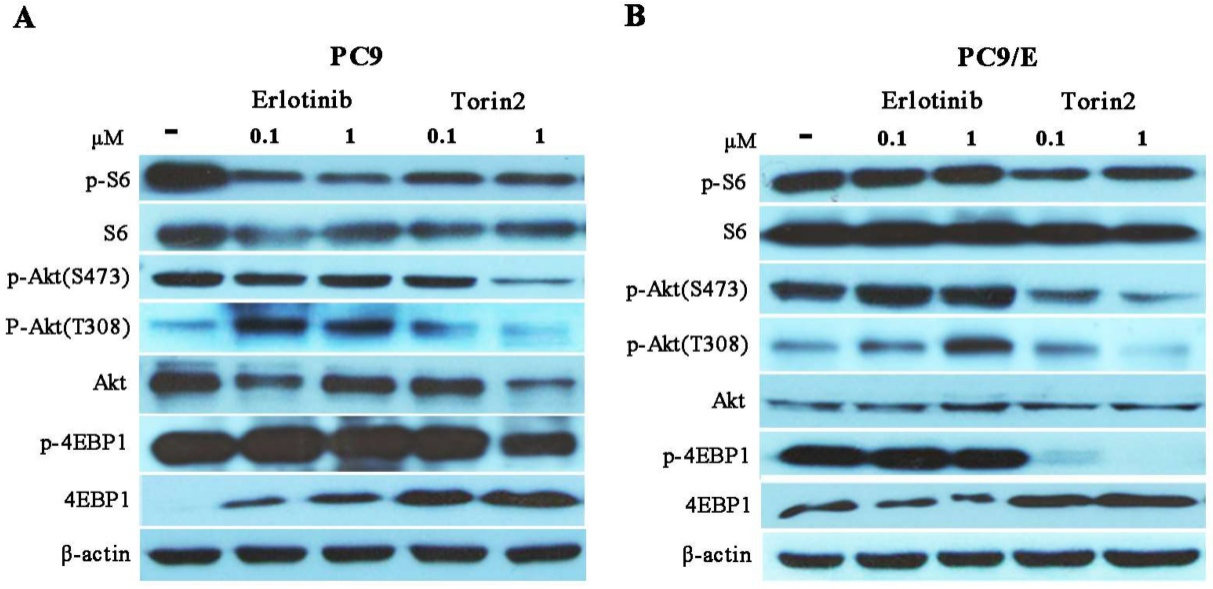
** Torin2 inhibited the growth of EGFR-TKI resistant cell by down-regulated negative-feedback loop of Akt/mTOR signaling pathway. (A)** Western blot analysis showed that level of phosphorylated S6 was reduced in PC9 cell treated with Erlotinib or Torin2, respectively. The level of phosphorylated Akt(T308) was activated by erlotinib, whereas Torin2 significantly diminished the phosphorylated Akt(T308) and Akt(S473) in a dose dependent manner. **(B)** In the PC9/E cell line, the expression of phosphorylated S6 and 4EBP-1 were obviously decreased in Torin2 treated group compared with erlotinib treatment. Torin2 also markedly down-regulated both phosphorylated Akt(S473) and Akt(T308) in PC9/E compared to erlotinib group.

To further investigate the possible mechanism for torin2 inhibited PC9/E cell growth, we investigated the mTOR signaling molecules in PC9 and PC9/E cells treated with erlotinib or torin2. In PC9 cells, erlotinib attenuated the phosphorylated S6 and failed to phosphorylated 4EBP1. In addition, the levels of phosphorylated Akt at S473 and T308 site were activated by erlotinib due to feedback loop of down-regulated level of phosphorylated S6. In contrast, torin2 attenuated phosphorylation of the mTORC1 substrates (pS6 and 4EBP1) and decreased phosphorylation of the mTORC2 substrates Akt (Ser473) in a concentration-dependent manner in PC9 cells (Figure 6A). Conversely, erlotinib failed to block the activated the phosphorylated S6, 4EBP1 and Akt in PC9/E cells. Moreover, torin2 not only inhibited phosphorylation of both mTORC1 substrates, pS6 and 4E-BP1, but also suppressed the phosphorylation of Akt (Ser473) and Akt (T308) in PC9/E cells (Figure 6B). Taken together, torin2 effectively inhibited cell viability of PC9/E cells through blocking a negative feedback regulation of mTOR signaling.

### Torin2 suppressed tumor growth and angiogenesis *in vivo*

**Figure 7:**
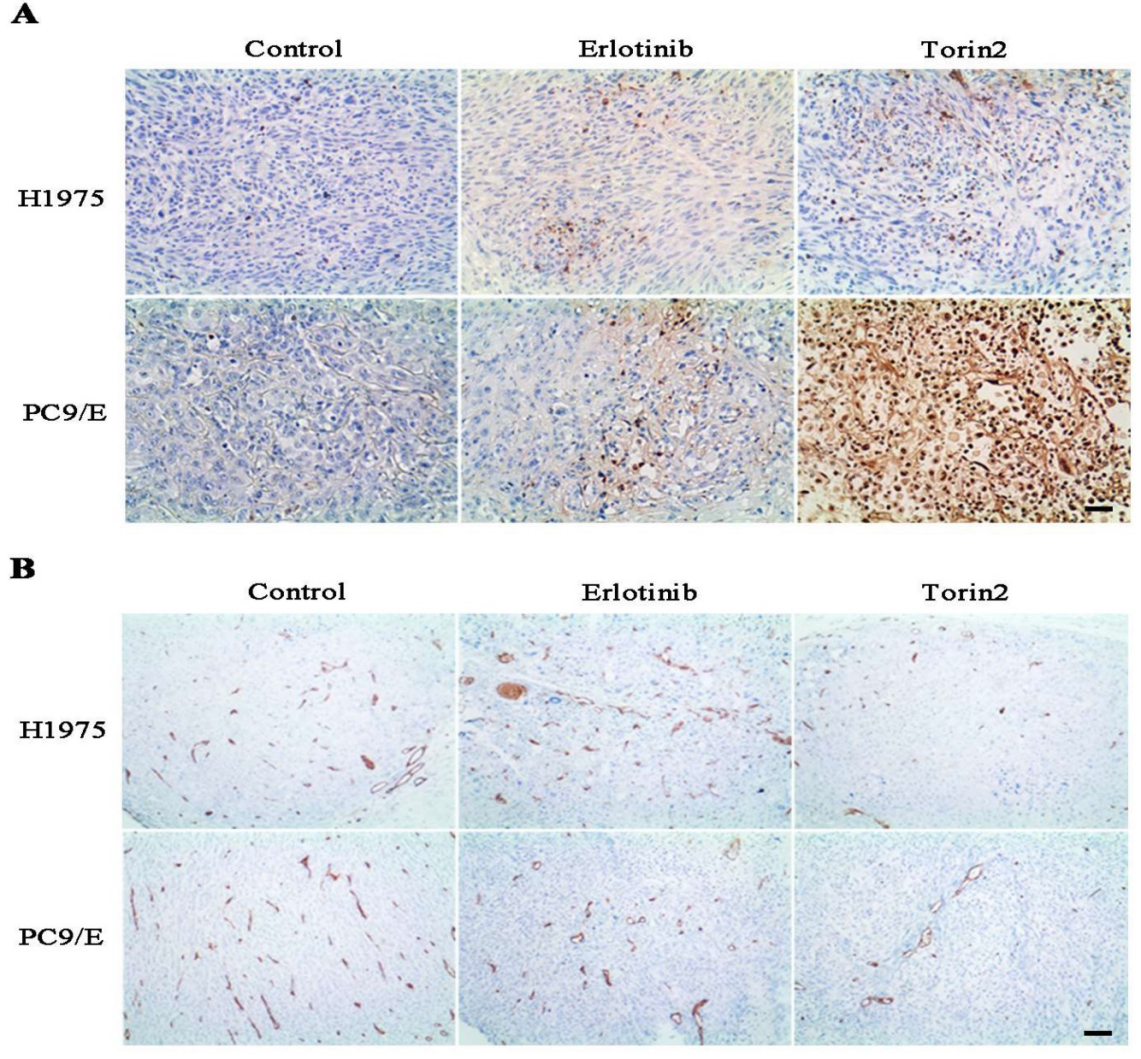
** Torin2 inhibited the tumor cell proliferation and angiogenesis* in vivo*. (A)** In the xenograft model, immunohistochemical analysis of TUNEL staining showed that Torin2 treatment obviously increased levels of apoptosis compared to the control and Erlotinib groups, either in subcutaneous injection of H1975 or PC9/E. **(B)** Tumor angiogenesis was analyzed by CD31 IHC staining from xenograft tumors tissue. CD31 in Torin2 treatment group was much less positive than erlotinib group and control, either from H1975 or PC9/E cell. Scale bar: 100 µm.

To further evaluate the effects of torin2 on EGFR-TKI resistant NSCLC cells growth *in vivo*, we measured the tumor growth and tumor angiogenesis from nude mice. H1975 cells and PC9/E cells were respectively xenografted into Nodscid or nude mice, immunohistochemical analysis displayed that the torin2 significantly promoted cell apoptosis, as reflected by more positive immunostaining for TUNEL staining (Figure 7A). Furthermore, the expression of CD31 was markedly reduced in the torin2-treated group than erlotinib-treatment group (Figure 7B). The number of tumor tissue vessels were decreased in torin2 treatment group. These results indicated that torin2 inhibited xenografted tumor growth through promoting apoptosis and inhibiting tumor angiogenesis.

## Discussion

In the study, we investigated that torin2 had an inhibitory effective on the EGFR-TKI resistant cell lines and sensitive cell lines. Torin2 is a second-generation ATP-competitive inhibitor, which is selective for mTORC1 inhibitor 27. Accumulated evidence indicated that torin2 treatment was successfully used in inhibiting tumor growth and metastasis of papillary thyroid carcinoma and anaplastic thyroid cancer 29, 33. But the effect of torin2 treatment on lung cancer patients was less well-known. Our data demonstrated that torin2 dramatically suppressed the proliferation of H1975, H1650 cell lines compared with erlotinib. Also, torin2 could effectively inhibit the viability of PC9/E cell line which is resistant to erlotinib. However, the mechanisms of torin2 in suppressing lung cancer cells, especially EGFR-TKI resistant lung cancer cells have not been clearly elucidated.

It is reported that torin2 caused dephosphorylation of rapamycin-sensitive mTORC1 substrates, such as S6K, rapamycin-insensitive mTORC1 substrates, such as 4EBP1, and mTORC2 substrates such as Akt (S473) 27. Previous review described that dysregulation of the Akt/mTOR signaling pathway was closely related to the tumor development, metastasis and apoptosis in lung cancer 20. Aberrant phosphorylated mTOR was detected in more than 70% of 110 NSCLC samples 8. mTOR is also a downstream target of EGFR signaling and primary clinical trials of mTOR inhibitors have demonstrated that the inhibitors may be effective against lung carcinoma 34, 35. Our results showed that torin2 dramatically inhibited the level of phosphorylated S6 and 4EBP-1 of mTORC1 signaling pathway, either in PC9/E or PC9. Torin2 also blocked mTORC1 in a dose-dependent manner in resistant NSCLC cell lines. We also found that erlotinib reduced the level of phosphorylated S6 protein, but the expression of phosphorylated Akt at T308 and S473 sites was enhanced due to negative feedback of mTORC1 signaling in PC9 cell. While torin2 obviously inhibited phosphorylated Akt (ser473) and mTORC1 activity, overcoming the feedback reactivation of Akt in PC9/E. It is suggested that torin2 may offer an advantage over rapamycin because torin2 inhibit the mTOR pathway and limit the mTORC1 pathway reactivation through abrogation of the S6K feedback loop. Moreover, torin2 significantly suppressed the tumor growth in PC9/E xenograft nude mice model through increasing apoptosis by TUNEL staining. Thus, the results demonstrated that torin2 had a strong inhibitory effect on EGFR-TKIs resistant lung cancer cells both *in vitro* and *in vivo.* The potential mechanism was associated with reducing the activity of Akt/mTOR signaling.

On the other hand, torin2 induced the overexpression of autophagy protein in EGFR-TKIs resistant NSCLC cell lines. Autophagy, as a cytoprotective response, has a dual role in oncogenesis and excessive autophagy may also be associated with tumor cell death 36-38. Recent studies indicated that erlotinib treatment increased LC3II expression, Atg-5/Atg12 conjugation and formation of AVO and p62 degradation in HCC827 and HCC4006 cells, which are sensitive to erlotinib 39. Autophagy was induced with erlotinib treatment in NSCLC cell lines, A549 and NCL-H1299 cells 40. Thus, it is indicated that autophagy was closely associated with EGFR-TKIs resistance. Han et al. demonstrated that EGFR-TKIs could activate cell autophagy to impair the sensitivity of lung cancer cells to targeted therapy by inhibiting the AKT-mTOR-p70S6K signaling pathway 41. AZD9291, a third generation of EGFR-TKIs, inhibited multiple lung cancer cells and induced autophagy in A549 and H1975 cells by inhibiting PI3K/Akt pathway 42. Our results demonstrated that erlotinib induced autophagy protein phosphatidylethanolamine-modified microtubule-associated protein light-chain 3 (LC3) and decreased phosphorylated S6 protein expression in PC9 cell, but not PC9/E cell. Also, torin2 triggered autophagy-mediated cell death in both PC9/E and H1975 cells by increasing the expression of LC3II/I. Increasing evidence has indicated that induced-autophagy was potential resistance mechanism to EGFR-TKIs. In current, the role of autophagy in EGFR-TKIs targeted therapy remains controversial. It is reported that induced autophagy was associated with EGFR-TKIs resistance in lung cancer and played a positive and negative dual role in promoting apoptosis in NSCLC 43. Some studies have indicated that inhibition of autophagy with chloroquine (CQ) enhanced the effect of erlotinib treatment in EGFR-mutated, but not in wild-type NSCLC cells 39. It demonstrated that autophagy inhibition had a beneficial effect on enhancing tumor cell death 44. Our data showed that higher LC3II expression was observed in PC9/E compared with PC9 cell line. The results indicated that induced-autophagy was related with EGFR-TKI resistance in lung cancer. In addition, erlotinib could induce autophagy in PC9 cell line and torin2 also markedly induced autophagy in PC9/E cell line. The results demonstrated that excessive autophagy could also lead to autophagic NSCLC cell death. Therefore, either positive or negative manipulation of autophagy, it could be an effective therapeutic strategy. Finally, torin2 inhibited the EGFR-TKI resistant NSCLC cells proliferation and decreased the numbers of tumor vessels in xenograft model. There was a significant difference in tumor size between torin2 treatment group and erlotinib group.

It is reported that torin2 also inhibited cellular activity against phosphatidylinositol 3-kinase-related kinase (PIKK) family kinases including ataxia telangiectasia-mutated kinase (ATM), ataxia telangiectasia-mutated, Rad3-related kinase (ATR) and DNA-PK, the inhibition of which sensitized cells to irradiation 45. Luo et al. showed that torin2 could enhance the radiosensitivity of breast cancer cells by downregulation the mTOR signaling and ATM phosphorylation 46. Moreover, torin2 inhibited lionizing radiation-induced DNA damage repair 47. It is reported Ku-0063794, a specific mTORC1 and mTORC2 inhibitor, could inhibit cell growth and induce a G1-cell-cycle arrest. The study demonstrated that Ku-0063794 was a more effective anti-cancer agent than rapamycin. Ku-0063794 and other mTOR inhibitors could effectively suppress protein synthesis resulting from overactivation of the PI3K/Akt pathways 48. Taken together, these studies indicated that torin2 could suppress the cells proliferation in lung cancer. However, it should be pointed out that torin2 could play an off-target role in carcinoma treatment. For instance, Heinzen D and colleagues reported that Torin2 not only inhibited the growth of cancer cells but also played protective effects on inducing oxygen and glucose consumption in hypoxia-induced cell death. The effect was induced by attenuating glycolytic metabolism 49. Thus, the study supports that combining torin2 with other treatments is very important for cancer treatments.

EGFR-TKI resistant mechanisms in NSCLC cells is so comprehensive that it remains to be further investigated. It is known that the third generation of EGFR-TKI, AZD9291, is highly selective against EGFR T790M‐mutant NSCLC. AZD9291 is still the first line treatment for EGFR T790M‐mutant NSCLC patients compared to other treatment for EGFR-TKI resistant patients. Additionally, the most common toxicity of torin2 is emaciation in mice treated with torin2. Thus, further studies need to confirm the clinical safety of torin2.

In conclusion, we found that torin2 exerted a strong anti-proliferative effect by suppressing negative feedback of the Akt/mTOR signaling pathway and inducing autophagic cell death. Taken together, torin2 may be considered to be an attractive and potential therapeutics for EGFR-TKIs resistant NSCLC.

## Abbreviations

NSCLC: non-small cell lung cancer
EGFR: The epidermal growth factor receptor
PI3K: phosphatidylinositol 3-kinase
TKIs: tyrosine kinase inhibitors
mTOR: mammalian target of rapamycin
LC3: phosphatidylethanolamine-modified microtubule-associated protein light-chain 3
